# Integrating Genotypic Data With Transcriptomic and Proteomic Data

**DOI:** 10.1002/cfg.135

**Published:** 2002-02

**Authors:** Denis C. Shields, Aisling M. O'Halloran

**Affiliations:** Department of Clinical Pharmacology Royal College of Surgeons in Ireland 123 St Stephen's Green Dublin 2 Ireland

## Abstract

Historically genotypic variation has been detected at the phenotypic level, at the metabolic level, and at the protein chemistry level. Advances in technology have allowed its direct visualisation at the level of DNA variation. Nevertheless, there is still an enormous interest in phenotypic, metabolic and protein property variability, since such variation gives insights into potential functionally important differences conferred by genetic variation. High-throughput transcriptomics and proteomics applied to different individuals drawn from a population has the potential to identify the functional consequences of genetic variability, in terms of either differences in expression of mRNA or in terms of differences in the quantities, pI(s) or molecular weight(s) of an expressed protein. Family studies can define the genetic component of such variation (segregation analysis) and with the genotyping of well-spaced markers can map the causative factors to broad chromosomal regions (linkage analysis). Association studies in the variant proteins have the greatest power to confirm the presence of *cis*-acting genetic variants. The most powerful study designs may combine elements of both family and association studies applied to proteomic and transcriptomic analyses. Such studies may provide appreciable advances in our understanding of the genetic aetiology of complex disorders.


**Comparative and Functional Genomics**
*Comp Funct Genom* 2002; **3**: 22–27.
**DOI:** 10.1002/cfg.135

**Conference Review**

### Integrating genotypic data with transcriptomic and proteomic data

Denis C. Shields* and Aisling M. O'Halloran
*Department of Clinical Pharmacology, Royal College of Surgeons in Ireland, 123 St Stephen's Green, Dublin 2, Ireland*

**Correspondence to: Department of Clinical Pharmacology, Royal College of Surgeons in Ireland, 123 St Stephen's Green, Dublin 2, Ireland. E-mail: dshields@rcsi.ie*

Received: 29 November 2001
Accepted: 4 December 2001
Published online: 2 January 2002

### Abstract

**Historically genotypic variation has been detected at the phenotypic level, at the metabolic level, and at the protein chemistry level. Advances in technology have allowed its direct visualisation at the level of DNA variation. Nevertheless, there is still an enormous interest in phenotypic, metabolic and protein property variability, since such variation gives insights into potential functionally important differences conferred by genetic variation. High-throughput transcriptomics and proteomics applied to different individuals drawn from a population has the potential to identify the functional consequences of genetic variability, in terms of either differences in expression of mRNA or in terms of differences in the quantities, pI(s) or molecular weight(s) of an expressed protein. Family studies can define the genetic component of such variation (segregation analysis) and with the genotyping of well-spaced markers can map the causative factors to broad chromosomal regions (linkage analysis). Association studies in the variant proteins have the greatest power to confirm the presence of *cis*-acting genetic variants. The most powerful study designs may combine elements of both family and association studies applied to proteomic and transcriptomic analyses. Such studies may provide appreciable advances in our understanding of the genetic aetiology of complex disorders. Copyright © 2002 John Wiley & Sons, Ltd.**

**Keywords:** **genotyping; transcriptomics; proteomics; microarray; segregation; linkage; association; Single Nucleotide Polymorphism**

### Background

For the past two decades much attention has been focused on detecting the genetic variation involved in monogenic disorders of major effect, such as cystic fibrosis [1,2] and retinitis pigmentosa [3]. These have been mapped to broad chromosomal regions by following the co-segregation in families of the disease with a limited panel of polymorphic markers located at intervals along human chromosomes (linkage analysis). For complex disorders such as cardiovascular and psychiatric disease there is a strong heritable component, however, this results from the accumulated small effects of many functional polymorphisms. Linkage analysis is not so powerful in these cases, since there is only a small increase in the marker co-segregation of a given polymorphism with the disease [4]. Even association studies may have limited power if the number of patients studied with a homogeneous phenotype is restricted. Thus, the scientific literature abounds with conflicting reports regarding the significance of associations between particular genetic variants and disease [5,6,7]. Association studies have the greater drawback that, until larger sample sizes and cheaper genotyping can justify genome-wide scans [4], they generally start with candidate loci. An absence of association of a given variant does not exclude that protein from a role in the disease (since many polymorphisms have no physiological significance). Conversely, the presence of a weak association does not prove that the protein is critical. Many of the risk factors associated with cardiovascular disease identified through association studies confer low risks (of the order of a 10% increase in risk) when meta-analysis of many studies is performed [8,9], which may largely explain the inability of many studies to detect significant

Copyright © 2002 John Wiley & Sons, Ltd.

associations. Polymorphisms common in the general population (whose study has changed the understanding of a disease process) have in the past been frequently initially detected through biochemical, rather than genetic approaches (eg., the coagulation Factor V Leiden variant [10]). The challenge going forward for complex diseases is to carry out genetic studies that provide novel and interesting insights into the biological processes rather than merely confirming what is known of the disease process from other studies. Since the links between the genotypic variants and the disease outcome are weak, the means to improve understanding is to collect information on the intermediate RNA, protein and metabolic mediators of risk between genotype and disease. Genotypic data has one crucial feature which makes it valuable in constructing causal models in complex disease: unlike the RNA, protein and metabolic phenotypes, the genotype is generally not modified by the disease process itself. Such causal models require genotypic information, disease status information, and the intermediate biomolecular information. The development of high-throughput technologies for genotyping, for studying many RNA species simultaneously (transcriptomics) and for studying many protein species simultaneously (proteomics) offers a powerful approach for the genetic dissection of complex disorders.

### Genetically determined variability in mRNA expression level

Studies of genetic variation in mRNA levels of genes are currently mainly limited to analyses of genetic variants in the regulatory regions of genes. The impact of such regulatory variants on the expression of the gene can be assessed by generating gene constructs and introducing them into experimental cellular systems. There is a growing literature of such information but no centralised database of such experimental findings, detailing the tissue origin of the cell line used and the relative levels of expression of the alternative variants. The physiological relevance of mRNA expression differences in cell systems to in vivo expression is usually not explored. However, it is also feasible to directly investigate mRNA level in relation to genotype. For example, the level of Angiotensin-1 converting enzyme mRNA was measured in kidney biopsies from 50 patients and correlated with genotypic differences in the well-studied insertion-deletion polymorphism in this gene [11]. Ex vivo mRNA analysis in cells cultured from a number of individuals provides another way to compare inter-individual variation in mRNA level with genotype [12]. This provides greater control of the conditions for handling and processing the RNA, although it is one step away from the true physiological context. cDNA microarrays have been used in comparing the expression pattern in patients with different disease-causing genotypes, for example in the comparison of cancer gene expression profiles between BRCA1 and BRCA2 carriers [13]. Such a comparison is looking at the downstream (*trans*) effect of a very large genotypic difference on gene expression. The challenge will be to identify less striking, but biologically important, associations between many possible genotypic variants and changes in mRNA expression, both in *cis* and in *trans* [14]. To date, microarray analyses have been most useful in cancer studies, where the very marked alterations in the co-ordinated expression of groups of genes lie well outside the margins of experimental error found with current microarray analyses.

### Genetically determined variability at the protein level

Proteomics is the surveying of a large number of proteins at once. Currently, the main technology is separation by two-dimensional gel electrophoresis, whose analytical capabilities have been accelerated in the last few years by rapid protein identification using mass spectrometry. This is likely to be routinely augmented in the future by more sensitive technologies. Here we give examples of some of the genetically determined factors identified in two-dimensional gels.

### Detection of genetically determined protein variation

Genetically determined variation may potentially be observed by whatever means proteins are studied. Anderson and Anderson [15] silver stained two-dimensional gels to observe a number of polymorphisms among the abundant proteins detectable on the gel. More specific analyses restrict the number of proteins observed, for example by immunoblotting against a single protein [16], or group of

Copyright © 2002 John Wiley & Sons, Ltd. 

proteins, such as spectrins [17], or Glutathione-S transferases [18].

### Classes of genetic variants

Genetic variants that have been analysed using 2-D gels include: (a) common polymorphisms, observed as protein variations between individuals drawn from a species, eg. in human serum [15], or in maize [19], (b) rare disease mutations [16], (c) somatic mutations observed in cancer cells [18], or (d) novel variants induced experimentally by mutagenesis [20].

### Nature of protein variability detectable on 2D gel

Variation may be in the quantity [19,20], size, or isoelectric point (pI) of the protein, or it may be whether or not the protein forms large molecular weight, higher-order protein complexes under controlled conditions of protein preparation [16].

### Action of genetic polymorphism on protein molecular phenotype

Genetic variants may act in *cis* (the genetic variation affecting the protein's appearance on the gel lies within the gene for the protein) or in *trans* (the genetic variation influencing the protein lies within another gene which influences the level of expression, or the post-translational modification, of the protein [19]). Linkage analysis can reveal whether the underlying variation maps to the chromosomal region of the gene that encodes the variant protein (*cis*), or whether it lies outside this region (*trans*) [19].

### Family/pedigree and association studies

### Segregation analysis of molecular phenotypes

Historically, genetic analyses of complex diseases in humans have often involved a segregation analysis of the disease condition to determine if its pattern of inheritance in families follows dominant, recessive, or polygenic models, or some mixture of the above, and to estimate the likelihood of disease given a particular genetic make-up (penetrance) as well as the likely frequency of the alleles in the population [21]. A simpler approach is to calculate the relative risk to a sibling of having the phenotype, compared to the risk of an unrelated control. However, this simpler method ignores whether the molecular variant is behaving in a recessive, dominant or dose-dependent manner, and may therefore be less powerful. Segregation analysis in pedigrees or families permits an estimation of the heritability of the molecular phenotype [14].

### Linkage analysis of molecular phenotypes

Linkage analysis [22] of a molecular phenotype offers the potential to determine if the factor underlying the variation lies within the chromosomal region of the gene that encodes the gene product, or outside it.

### Association studies

Association studies simply ask: in a group of unrelated individuals, is a certain genotype more frequent with a certain phenotype. The phenotype may be a comparison of cases with controls (such as persons with a protein variant of a particular mass and pI compared to controls who lack this variant), or alternatively a study of a quantitative variable (such as protein or RNA level) within a group.

### Study design and power

Segregation, linkage and association analyses provide the three basic tools whereby the links between genotype and a molecular phenotype can be explored. Each may be of use on its own, since a protein variant which segregates strongly in a Mendelian fashion may be a marker for important disease processes measurable in the clinical phenotype. While a strong Mendelian pattern of inheritance may be more usually consistent with a *cis* effect within the protein, segregation analysis alone cannot determine if the genetic variability lies within the gene encoding the protein. Linkage analysis will determine the broad chromosomal location of the genetic variant underlying a molecular phenotype. Linkage analysis has the advantage that the genome-wide scan of well-spaced, informative markers, once performed, can be used to potentially map all the molecular variants, which may each be analysed as quantitative trait loci [14] or subjected to combined segregation and linkage analyses [23]. Linkage analysis may be of particular interest in the detection of protein–protein interactions, since *trans*-acting genetic factors modifying a protein's pI, molecular weight or level may be broadly mapped to a chromosomal region.

Copyright © 2002 John Wiley & Sons, Ltd. 

However, in order to increase the resolution of this linkage analysis to reasonably narrow chromosomal regions with smaller numbers of candidate genes, quite a large panel of families may be required. Association studies are quicker and easier, but they may be best carried out after an initial segregation analysis, to prevent extensive negative studies of variations that have little or no strong genetic basis. Association studies can directly assess whether known polymorphisms within the variant protein can account for that polymorphism. Direct observation of a single causal variant is always more powerful than linkage analysis. Linkage analysis is likely to be more powerful than whole-genome association studies for molecular variants with high heritability [4]. Thus, association studies may be the approach of choice for *cis* effects, while linkage analysis is appropriate for detecting any possible *trans* effects. The best study design may combine all three approaches.

### Further integration of genetic, transcriptomic and proteomic data

Study design and subsequent interpretation may be conditioned on a priori biological information. For example, polymorphisms analysed may be initially restricted to those which are more likely to be of functional importance [24]. *Trans*-acting changes may affect large numbers of genes (eg., genetic variation in a critical signalling pathway could influence a co-ordinated increase in the levels of a group of genes). Interpretation of biological clusters of changes may rely on a priori classification of gene functions based on general classifications (eg., see www.geneontology.org), or on other means of clustering genes [25] (co-occurrence in species; co-expression in tissues; experimentally observed protein–protein interactions; clusters of homologous proteins; automated interpretation of databases of scientific literature). This broader level of

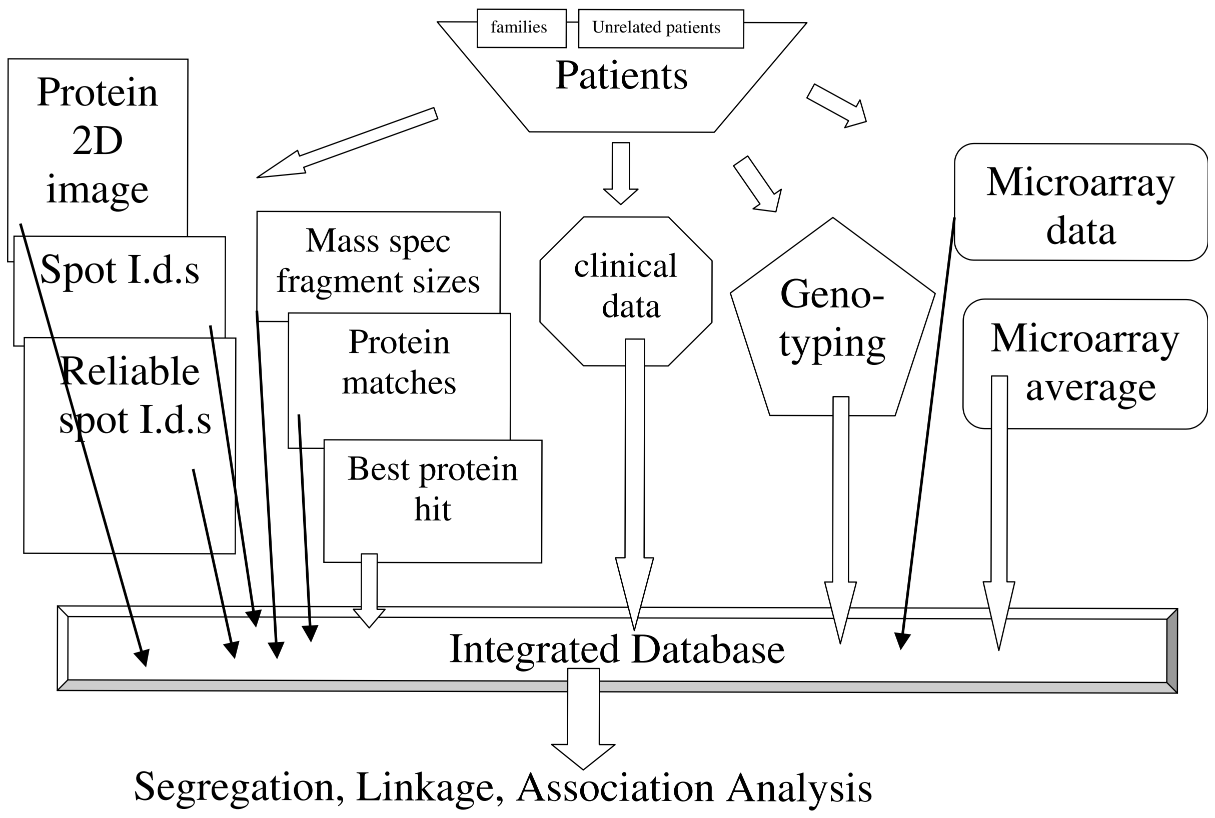

**Figure 1.** Representation of data flow in a study of human disease integrating genotypic data with proteomic and expression analysis. Block arrows represent the major flow of data, thin arrows indicate the possible linkages between the analysis database and the underlying raw data

Copyright © 2002 John Wiley & Sons, Ltd. 

data integration is not unique to the specific questions of relating genotypic variation to other molecular variation, but will need to be addressed. Integration of the clinical data from each patient in terms of genes, proteins and messages itself represents a reasonably complex task depending on how much the final analytical database takes forward from the raw experimental data, and how much it relies on summaries across experiments and database searches (Figure 1).

### Conclusions

Integration of genotypic, proteomic and transcriptomic data is technically feasible. Over the past three decades numerous analyses of genetic variability underlying mRNA and protein variation, usually restricted to a relatively limited number of gene products, have accumulated. Whilst the genotyping component is relatively straightforward, the challenge is to scale such studies up for high-throughput analyses that can measure the protein and mRNA products at the required sensitivity. Such integrated studies with well-measured molecular and clinical phenotypes in humans have the potential to transform our understanding of the genetic basis of complex disorders. However, initial advances may come via the application of these methods to transgenic and model organisms [14,26], where well designed experimental crosses between diverged strains appear to be highly informative in detecting protein expression differences with a genetic aetiology [19].

### Acknowledgement

This work was supported by the Higher Education Authority (Ireland) through its funding to the Biopharmaceutical Sciences Network.

### References

1. Kerem B, Rommens JM, Buchanan JA, *et al.* 1989. Identification of the cystic fibrosis gene: genetic analysis. *Science* **245**: 1073–1080.
2. Riordan JR, Rommens JM, Kerem B, *et al.* 1989. Identification of the cystic fibrosis gene: cloning and characterization of complementary DNA. *Science* **245**: 1066–1073.
3. Farrar GJ, Kenna P, Jordan SA, *et al.* 1992. Autosomal dominant retinitis pigmentosa: a novel mutation at the peripherin/RDS locus in the original 6p-linked pedigree. *Genomics* **14**: 805–807.
4. Risch N, Merikangas K. 1996. The future of genetic studies of complex human diseases. *Science* **273**: 1516–1517.
5. Kirov G, Jones I, McCandless F, Craddock N, Owen MJ. 1999. Family-based association studies of bipolar disorder with candidate genes involved in dopamine neurotransmission: DBH, DAT1, COMT, DRD2, DRD3 and DRD5. *Mol Psychiatry* **4**: 558–565.
6. Gurlek A, Gulec S, Karabulut H, *et al.* 2000. Relation between the insertion/deletion polymorphism of the angiotensin I converting enzyme gene and restenosis after coronary stenting. *J Cardiovasc Risk* **7**: 403–407.
7. Koch W, Kastrati A, Mehilli J, Bottiger C, von Beckerath N, Schomig A. 2000. Insertion/deletion polymorphism of the angiotensin I-converting enzyme gene is not associated with restenosis after coronary stent placement. *Circulation* **102**: 197–202.
8. Di Castelnuovo A, de Gaetano G, Donati MB, Iacoviello L. 2001. Platelet glycoprotein receptor IIIa polymorphism PIA1/PIA2 and coronary risk: a meta-analysis. *Thromb Haemost* **85**: 626–633.
9. Keavney B, McKenzie C, Parish S, *et al.* 2000. Large-scale test of hypothesised associations between the angiotensin-converting-enzyme insertion/deletion polymorphism and myocardial infarction in about 5000 cases and 6000 controls. International Studies of Infarct Survival (ISIS) Collaborators. *Lancet* **355**: 434–442.
10. Lee R. 2001. Factor V Leiden: a clinical review. *Am J Med Sci* **322**: 88–102.
11. Mizuiri S, Hemmi H, Kumanomidou H, *et al.* 2001. Angiotensin-converting enzyme (ACE) I/D genotype and renal ACE gene expression. *Kidney Int* **60**: 1124–1130.
12. Mark LL, Haffajee AD, Socransky SS, *et al.* 2000. Effect of the interleukin-1 genotype on monocyte IL-1beta expression in subjects with adult periodontitis. *J Periodontal Res* **35**: 172–177.
13. Hedenfalk I, Duggan D, Chen Y, *et al.* 2001. Gene-expression profiles in hereditary breast cancer. *N Engl J Med* **344**: 539–548.
14. Jansen RC, Nap JP. 2001. Genetical genomics: the added value from segregation. *Trends Genet* **17**: 388–391.
15. Anderson L, Anderson NG. 1977. High resolution two-dimensional electrophoresis of human plasma proteins. *Proc Natl Acad Sci U S A* **74**: 5421–5425.
16. Tiranti V, Corona P, Greco M, *et al.* 2000. A novel frameshift mutation of the mtDNA COIII gene leads to impaired assembly of cytochrome c oxidase in a patient affected by Leigh-like syndrome. *Hum Mol Genet* **9**: 2733–2742.
17. DiPaolo BR, Speicher KD, Speicher DW. 1993. Identification of the amino acid mutations associated with human erythrocyte spectrin alpha II domain polymorphisms. *Blood* **82**: 284–291.
18. Schisselbauer JC, Hogan WM, Buetow KH, Tew KD. 1992. Heterogeneity of glutathione S-transferase enzyme and gene expression in ovarian carcinoma. *Pharmacogenetics* **2**: 63–72.
19. de Vienne D, Maurice A, Josse JM, Leonardi A, Damerval C. 1994. Mapping factors controlling genetic expression. *Cell Mol Biol* **40**: 29–39.
20. Champion KM, Cook RJ, Tollaksen SL, Giometti CS. 1994. Identification of a heritable deficiency of the folate-dependent enzyme 10-formyltetrahydrofolate dehydrogenase in mice. *Proc Natl Acad Sci U S A* **91**: 11338–11342.

Copyright © 2002 John Wiley & Sons, Ltd. 

21. Morton NE. 1982. *Outline of Genetic Epidemiology*. S. Karger: Basel
22. Ott J. 1999. *Analysis of Human Genetic Linkage*. 3rd edition. Johns Hopkins University Press: Baltimore.
23. Shields DC, Ratanachaiyavong S, McGregor AM, Collins A, Morton NE. 1994. Combined segregation and linkage analysis of Graves disease with a thyroid autoantibody diathesis. *Am J Hum Genet* **55**: 540–554.
24. Chasman D, Adams RM. 2001. Predicting the functional consequences of non-synonymous single nucleotide polymorphisms: structure-based assessment of amino acid variation. *J Mol Biol* **307**: 683–706.
25. Eisenberg D, Marcotte EM, Xenarios I, Yeates TO. 2000. Protein function in the post-genomic era. *Nature* **405**: 823–826.
26. Ziv M, de Vienne D. 2000. Proteomics: a link between genomics, genetics and physiology. *Plant Mol Biol* **44**: 575–580.

Copyright © 2002 John Wiley & Sons, Ltd. 

## References

[PDF_00420] Anderson L., Anderson N. G. (1977). High resolution two-dimensional electrophoresis of human plasma proteins.. Proc Natl Acad Sci U S A.

[PDF_00437] Champion K. M., Cook R. J., Tollaksen S. L., Giometti C. S. (1994). Identification of a heritable deficiency of the folate-dependent enzyme 10-formyltetrahydrofolate dehydrogenase in mice.. Proc Natl Acad Sci U S A.

[PDF_00451] Chasman D., Adams R. M. (2001). Predicting the functional consequences of non-synonymous single nucleotide polymorphisms: structure-based assessment of amino acid variation.. J Mol Biol.

[PDF_00396] Di Castelnuovo A., de Gaetano G., Donati M. B., Iacoviello L. (2001). Platelet glycoprotein receptor IIIa polymorphism PLA1/PLA2 and coronary risk: a meta-analysis.. Thromb Haemost.

[PDF_00427] DiPaolo B. R., Speicher K. D., Speicher D. W. (1993). Identification of the amino acid mutations associated with human erythrocyte spectrin alpha II domain polymorphisms.. Blood.

[PDF_00455] Eisenberg D., Marcotte E. M., Xenarios I., Yeates T. O. (2000). Protein function in the post-genomic era.. Nature.

[PDF_00376] Farrar G. J., Kenna P., Jordan S. A., Kumar-Singh R., Humphries M. M., Sharp E. M., Sheils D., Humphries P. (1992). Autosomal dominant retinitis pigmentosa: a novel mutation at the peripherin/RDS locus in the original 6p-linked pedigree.. Genomics.

[PDF_00387] Gürlek A., Güleç S., Karabulut H., Bokesoy I., Tutar E., Pamir G., Alpman A., Toydemir R., Aras O., Oral D. (2000). Relation between the insertion/deletion polymorphism of the angiotensin I converting enzyme gene and restenosis after coronary stenting.. J Cardiovasc Risk.

[PDF_00415] Hedenfalk I., Duggan D., Chen Y., Radmacher M., Bittner M., Simon R., Meltzer P., Gusterson B., Esteller M., Kallioniemi O. P. (2001). Gene-expression profiles in hereditary breast cancer.. N Engl J Med.

[PDF_00418] Jansen R. C., Nap J. P. (2001). Genetical genomics: the added value from segregation.. Trends Genet.

[PDF_00400] Keavney B., McKenzie C., Parish S., Palmer A., Clark S., Youngman L., Delépine M., Lathrop M., Peto R., Collins R. (2000). Large-scale test of hypothesised associations between the angiotensin-converting-enzyme insertion/deletion polymorphism and myocardial infarction in about 5000 cases and 6000 controls. International Studies of Infarct Survival (ISIS) Collaborators.. Lancet.

[PDF_00370] Kerem B., Rommens J. M., Buchanan J. A., Markiewicz D., Cox T. K., Chakravarti A., Buchwald M., Tsui L. C. (1989). Identification of the cystic fibrosis gene: genetic analysis.. Science.

[PDF_00382] Kirov G., Jones I., McCandless F., Craddock N., Owen M. J. (1999). Family-based association studies of bipolar disorder with candidate genes involved in dopamine neurotransmission: DBH, DAT1, COMT, DRD2, DRD3 and DRD5.. Mol Psychiatry.

[PDF_00392] Koch W., Kastrati A., Mehilli J., Böttiger C., von Beckerath N., Schömig A. (2000). Insertion/deletion polymorphism of the angiotensin I-converting enzyme gene is not associated with restenosis after coronary stent placement.. Circulation.

[PDF_00411] Mark L. L., Haffajee A. D., Socransky S. S., Kent R. L., Guerrero D., Kornman K., Newman M., Stashenko P. (2000). Effect of the interleukin-1 genotype on monocyte IL-1beta expression in subjects with adult periodontitis.. J Periodontal Res.

[PDF_00408] Mizuiri S., Hemmi H., Kumanomidou H., Iwamoto M., Miyagi M., Sakai K., Aikawa A., Ohara T., Yamada K., Shimatake H. (2001). Angiotensin-converting enzyme (ACE) I/D genotype and renal ACE gene expression.. Kidney Int.

[PDF_00373] Riordan J. R., Rommens J. M., Kerem B., Alon N., Rozmahel R., Grzelczak Z., Zielenski J., Lok S., Plavsic N., Chou J. L. (1989). Identification of the cystic fibrosis gene: cloning and characterization of complementary DNA.. Science.

[PDF_00380] Risch N., Merikangas K. (1996). The future of genetic studies of complex human diseases.. Science.

[PDF_00431] Schisselbauer J. C., Hogan W. M., Buetow K. H., Tew K. D. (1992). Heterogeneity of glutathione S-transferase enzyme and gene expression in ovarian carcinoma.. Pharmacogenetics.

[PDF_00448] Shields D. C., Ratanachaiyavong S., McGregor A. M., Collins A., Morton N. E. (1994). Combined segregation and linkage analysis of Graves disease with a thyroid autoantibody diathesis.. Am J Hum Genet.

[PDF_00423] Tiranti V., Corona P., Greco M., Taanman J. W., Carrara F., Lamantea E., Nijtmans L., Uziel G., Zeviani M. (2000). A novel frameshift mutation of the mtDNA COIII gene leads to impaired assembly of cytochrome c oxidase in a patient affected by Leigh-like syndrome.. Hum Mol Genet.

[PDF_00458] Zivy M., de Vienne D. (2000). Proteomics: a link between genomics, genetics and physiology.. Plant Mol Biol.

[PDF_00434] de Vienne D., Maurice A., Josse J. M., Leonardi A., Damerval C. (1994). Mapping factors controlling genetic expression.. Cell Mol Biol (Noisy-le-grand).

